# Study on the Wear Mechanism of a Diamond AFM Tip During Scribing of a Single-Crystal Silicon

**DOI:** 10.3390/mi17030344

**Published:** 2026-03-12

**Authors:** Xinyue Lin, Litao Qi, Jinguo Han

**Affiliations:** 1 School of Mechanical Engineering, Heilongjiang University of Science and Technology, Harbin 150022, China; 2 School of Mechanical Engineering, Shandong University of Technology, Zibo 255000, China; hankeyee@163.com

**Keywords:** atomic force microscopy scribing, diamond probe, tip wear mechanism, molecular dynamics simulation, adhesive wear

## Abstract

To elucidate the wear mechanisms of diamond AFM tips during nanoscale scribing of single-crystal silicon, this study combines controlled experiments with atomistic molecular dynamics (MD) simulations. Scribing tests were conducted under systematically varied bias current, scribing speed, and scribing distance. Tip morphology evolution was quantitatively characterized. Concurrently, a three-dimensional MD model reproduced probe–silicon interactions to analyze bond breaking, atomic detachment, and structural transformation at the atomic scale. The results show that increasing current, speed, and distance significantly accelerate tip blunting. Simulations reveal a progressive transition in deformation behavior from elastic response to atomic attrition, plastic damage, brittle cracking, and catastrophic fracture as indentation depth increases, and cluster analysis establishes a quantitative correlation between process parameters and wear severity. This integrated experimental simulation framework provides mechanistic insight into diamond tip degradation and offers quantitative guidance for improving probe durability and process reliability in AFM-based nanofabrication.

## 1. Introduction

Atomic force microscopy (AFM) is widely used in ultra-precision machining, with applications in mechanical scribing [1], thermal machining [2], and electrochemical manufacturing [3]. Diamond probes are often employed for AFM-based scribing of hard materials such as single-crystal silicon [4] and silicon carbide [5] due to their high hardness and small tip radius. They are also used in the fabrication of optical components. The tip radius directly affects machining resolution, depth, and subsurface damage. Therefore, tip wear is a key factor in determining final machining accuracy [6,7,8].

Several studies have reviewed AFM probe wear, focusing on both simulations and experiments. Khurshudov et al. [9] report that silicon nitride AFM tips worn under 10 nN loads could still maintain imaging resolution. They attribute this to the formation of multiple sharp asperities induced by cyclic fatigue. Haitjema et al. [10] investigated the wear behavior of Si_3_N_4_ tips during prolonged contact-mode scanning. They observed an initial stage of surface passivation, followed by localized brittle spallation that formed submicron pits. Sample-derived debris was also found adhered to the tip surface, suggesting adhesive transfer. High stress near the apex induced an elastic–plastic transition, leading to confined plastic deformation and delamination of thin surface layers. Repeated scanning subsequently caused subsurface microcracks and gradual tip blunting. Chung et al. [11] used atomic-resolution HRTEM to examine monocrystalline silicon AFM tips after scanning. They observed a spontaneously formed amorphous silica layer with non-stoichiometric Si:O ratios on the tip surface. Comparative experiments in air and in a nitrogen atmosphere showed that oxidative reactions, rather than mechanical abrasion, are primarily responsible for tip mass loss under typical AFM conditions. This indicates that oxidation is the dominant wear mechanism.

Molecular dynamics (MD) simulation as an effective tool can provide the atomic-level machining evolution process, thereby providing important support for revealing the deformation mechanism of materials; it has been widely applied in ultra-precision processing. MD studies of nano-scratching were conducted by Liu et al. [12] to investigate the material removal mechanism and subsurface damage formation during nano-scratching of the gold–platinum. Nano-grinding simulation models were conducted by Wang et al. [13] to systematically analyze the material removal mechanisms on both the C-face and Si-face through a comprehensive comparison with the experiments. These results show that MD is not intended to replace experiments, but rather to rigorously establish a link between macroscopic phenomenon and its underlying mechanism atomistic basis through calculation. Additionally, a classical MD simulation was carried out by Fan et al. [14] to find that there was a new mode of wear not revealed in the traditional study before nanomachining of GaAs. At the apex of local heating on the diamond tip, there was a reversible transformation from sp^3^ hybrid orbitals to sp^2^ hybrid orbitals, and thus surface graphitisation occurred. Li et al. [15] used atomistic simulations and AFM experiments to study silicon tip wear under high-frequency tapping on single-crystal Si substrates. They found that permanent tip deformation is mainly caused by the nucleation and lateral growth of amorphous silicon domains, not by plastic flow or fracture. The wear process first showed a rapid transient stage and then entered a quasi-steady stage. Xi et al. [16] simulated diamond-based nanoscribing of 4H-SiC using MD. They investigated how normal load, sliding velocity, and tip geometry influence material removal, subsurface defects, and dislocation networks. The simulations revealed atomic-scale mechanisms that determine the nanoscale machinability of SiC. Goel et al. [17] modeled diamond turning of 3C-SiC with MD simulations and analyzed tool wear evolution. Their results show that diamond probe degradation is mainly due to thermally assisted graphitization caused by localized heating and abrasive contact. This process involves a transition from ordered sp^3^ bonding to disordered sp^2^ coordination, rather than purely mechanical wear. Most existing studies have used simulations to investigate silicon probes and workpiece materials such as silicon carbide and single-crystal copper. Oxidation wear [18], fracture wear [19], and atomic attrition wear [20] have been identified as the main wear mechanisms for silicon probes. For diamond tools, chemical wear is dominant [21].

Despite significant advances in simulations and experimental characterization, research on the wear mechanisms of diamond probes during scribing of single-crystal silicon remains limited. In particular, no systematic study has addressed how different machining parameters influence probe wear.

This work investigates the wear behavior of diamond-coated AFM probes during nanoscale scribing of monocrystalline silicon by combining experiments with MD simulations. This study is novel for its synergistic combination of a systematic quantitative parametric experiment and MD simulations to uncover the nanoscale wear mechanisms of AFM tips. In the experimental phase, precision scribing trials were performed on an AFM system while independently tuning key operational parameters—applied normal load (often correlated with probe current in conductive-AFM configurations), penetration depth, and lateral scan speed—to isolate their individual and coupled influences on probe degradation. Post-test probe wear was quantitatively characterized using high-resolution digital microscopy. In parallel, a three-dimensional MD model was developed to simulate probe–sample interfacial interactions and material removal processes under the same parametric conditions, enabling atomistic-level analysis of wear initiation and progression. These findings help clarify wear mechanisms and provide guidance for improving diamond probe durability and process reliability in AFM-based micro- and nanofabrication.

## 2. Materials and Methods

### 2.1. Test Equipment

**Figure 1:**
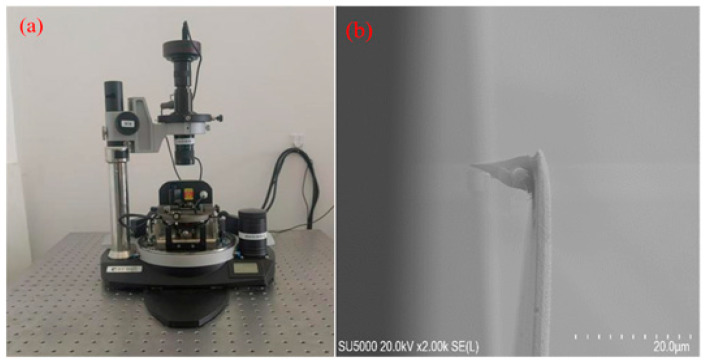
(**a**) NT-MDT atomic force microscope; (**b**) DCP20 diamond probe.

The experiments were conducted using an NTEGRA platform AFM (NT-MDT Company, Zelenograd, Moscow). A diamond probe (DCP20, TipsNano, Tallinn, Estonia) was used to scribe a 10 mm × 10 mm × 0.6 mm single-crystal silicon wafer with [100] crystallographic direction and (100) crystallographic plane. Probe wear morphology and tip radius measurements were carried out using a digital optical microscope (OLYMPUS DSX1000, Olympus Corporation, Tokyo, Japan). The digital optical microscope and probe are shown in Figure 1.

Tip radius measurements were performed using an OLYMPUS DSX1000 digital optical microscope at 100× magnification. Considering the potential uneven wear on the outer edge of the tip, five high-resolution images were collected for each probe; at different angles along the central axis of the tip, four radial sections could be reconstructed using these images. Edge detection was performed automatically using the microscope’s built-in algorithm, which employs a fixed grayscale threshold to distinguish the probe outline from the background. For each probe, the final reported radius is an average over the five independent angular measurements. At this time, it includes experimental repeatability deviation and an additional geometric asymmetry component caused by wear irregularity on the probe surface.

**Figure 2:**
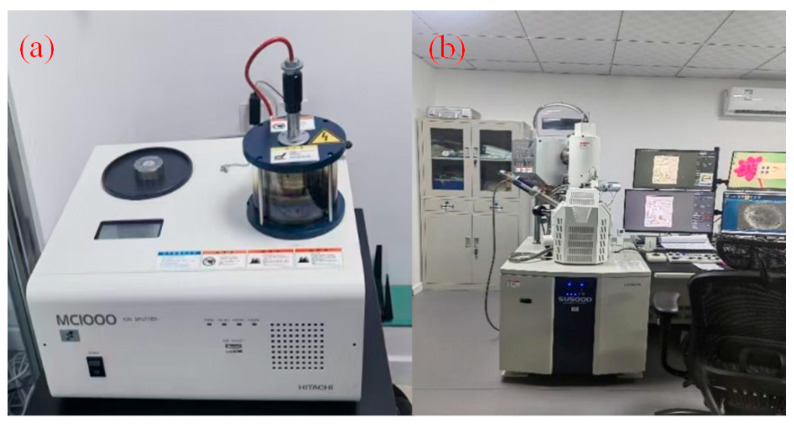
(**a**) Gold sputtering system (**b**) SEM imaging setup.

To improve the reliability of wear data, four independent manual scribings were conducted under the same conditions. After each test, the worn probe tips were inspected with a field-emission scanning electron microscope (FE-SEM, Su5000, Hitachi High-Tech Corporation, Tokyo, Japan). Before imaging, all probes were coated with a thin layer of gold using a sputter coater (MC1000, Hitachi High-Tech Corporation, Tokyo, Japan) for its accurate measurement. The facilities used are shown in Figure 2.

### 2.2. Test Method

#### 2.2.1. Current Control and Load Calibration

To establish a quantitative relationship between the electrical control parameters and the mechanical response of the diamond probe during nanoscratching, calibration was performed prior to the experiments. In this system, when applying a control current to the tip, its corresponding reaction force can be obtained through Equation (1) as follows:

(1) *F* = setpoint × sensitivity × *K.*

Setpoint refers to the current value of charge (in nA) preset in scratching, and it serves as the electrical signal input driving mechanical force. All scribing experiments were carried out under the condition that contacted objects were contacted and realized feedback through cantilever deflection. The current was simultaneously measured as a conductive-AFM signal but was not used as the feedback parameter. Sensitivity was calibrated experimentally using the single-point force–distance curve method on a rigid reference sample (silicon dioxide, SiO_2_) supplied by NT-MDT. The inverse slope of the retraction curve indicated a sensitivity of 13.2 ± 0.3 nm/nA. The spring constant of the microcantilever was determined by the integrated Sader_Normal tool in the NTEGRA 3.4.0 system. To reduce the amount of calculation and error, the average of five measurements was taken, and the result was *K* = 35.48 ± 0.5 N/m.

**Figure 3:**
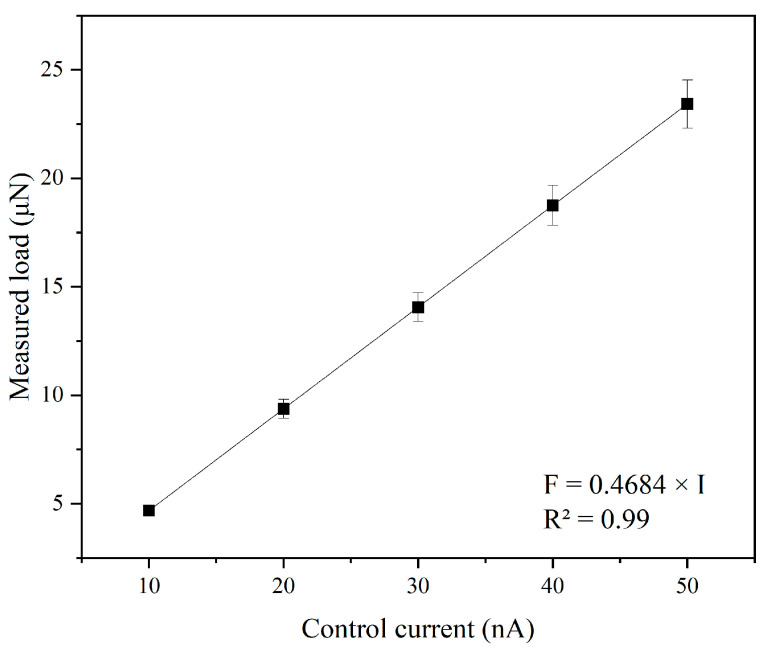
Calibration curve of normal force versus control current. Data points represent mean values, error bars indicate standard deviation. The solid line shows the linear fit.

After calibration, the values were converted through the setpoint conversion to a normal force value using Equation (1). The calibration curve of a linear relationship between current and force is shown in Figure 3. The data point represents the calculated force of each current, and there are error bars showing propagated uncertainty. Linear fitting gives an equation of *F* = 0.4684*I* (R^2^ = 0.99), where *F* is in μN, and *I* is in nA. Relationship data between the control current and actual load is shown in Table 1. To evaluate the stability under long scribing, the current was continuously adjusted at a constant force over 300 μm scribing distance. The drift of less than 5% could be tolerated, as there would be a slight increase in contact area caused by the tip wear, which does not affect the parametric trend analysis.

#### 2.2.2. Test Protocol

For all scribing experiments, contact mode was employed. Prior to testing, the correlation between control current and indentation depth was calibrated as discussed above. Detailed experimental conditions are listed in Table 2. Wear characteristics of the probes were observed with a digital optical microscope. For each experimental group, the average tip radius after wear was derived from five measured values.

### 2.3. Molecular Dynamics Simulation

#### 2.3.1. Model Development

**Figure 4:**
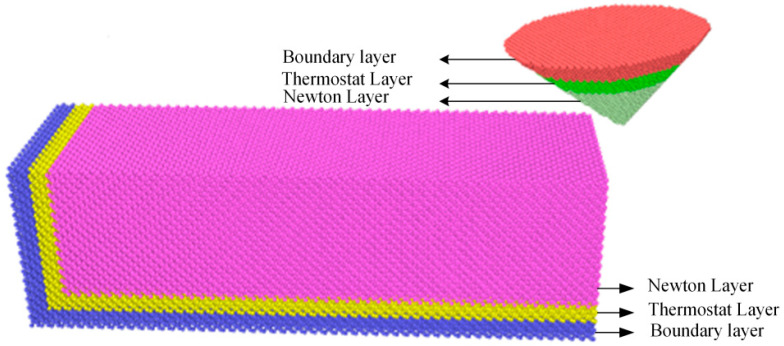
Simulation model.

In the simulation model, the probe was made of diamond (carbon atoms). The diamond probe has a cubic structure and a lattice constant of 3.57 Å. The probe is conical (height: 50 Å, top diameter: 50 Å, tip diameter: effectively 0 Å) and contains 23,302 carbon atoms. The parameter table is shown in Table 3. The diamond probe was not treated as a rigid body. Its atomic region is split into three layers: a boundary layer, a thermostat layer maintained at 298 K, and a Newtonian layer. The Newtonian layer is the main computational region where wear occurs. The MD model is shown in Figure 4.

This study uncovers the underlying wear dynamics of diamond-coated AFM probes during nanoscale mechanical scribing of single-crystal silicon, integrating high-precision experiments with atomistic-scale simulations. Scribing was executed on an AFM platform with precise, independent control over three critical process variables: bias current, penetration depth, and lateral scan speed. Tip morphology changes were tracked in situ and quantified post-scribing using high-magnification optical imaging. A three-dimensional molecular dynamics model was adopted to reveal the mechanism of AFM tip wear. It should be emphasized that MD simulations and experimental characterizations inherently differ in spatial scale, temporal resolution, and damage criteria. However, MD is an effective approach to reveal the deformation mechanism from the atomic perspective, which has been widely adopted in the ultraprecision machining field [12,13,14,15,16,17]. In this study, MD simulations are primarily employed to reveal the AFM tip wear mechanism at the atomic scale, serving as a complementary tool for mechanistic interpretation and trend analysis rather than for direct replication of experimental wear results.

By correlating experimental wear trends with simulated atomic trajectories, this work identifies the key factors governing tip degradation and provides practical guidance for improving tip durability and patterning accuracy in AFM-driven nanofabrication. The parameter settings for the monocrystalline silicon samples are shown in Table 4.

#### 2.3.2. Simulation Parameters for the Diamond Probe Scratch Test

The simulation employs the Tersoff potential function for constraints. The Tersoff potential function is extensively utilized in various covalent-bond solids and molecules. Diamond, a hard crystalline mineral formed by carbon atoms through covalent bonds, has atomic interactions that are highly suitable for description using the Tersoff potential function. Fan et al. [14] carried out MD simulations to study the wear process of AFM diamond probes on GaAs. They used the Tersoff potential function to constrain C–C interactions and ultimately concluded that cutting heat is the main cause of diamond probe wear.

Li et al. [15] conducted wear tests using silicon probes in the AFM tapping mode, with the etched materials being aluminum, copper, and silicon. In the silicon probe, the interactions between silicon atoms and between the probe and the silicon substrate material were constrained using the Tersoff potential function. Through MD analysis, significant stress gradients were obtained, which facilitated the transformation into a non-crystalline state. The geometric shape of the probe had a significant impact on the wear mode and deflection direction.

Alireza et al. [22] used the modified Tersoff potential function to increase the truncation coefficient to improve the properties of amorphous carbon. They proposed that the Tersoff potential function can be applied to the entire carbon material field, including graphite and diamond materials. The Tersoff potential function has achieved extensive success in the properties of carbon materials such as diamonds.

Erhart and Albe [23] developed a Tersoff parameterization specifically for C–Si interactions, which was validated across diverse configurations from dimers to bulk phases. This potential is employed in the present simulations to realistically capture atomic detachment and reattachment at the diamond–silicon interface during wear.

The simulated indentation depths were 10 Å, 15 Å, 20 Å, 25 Å, and 30 Å. Probe wear was simulated at scribing speeds of 10 m/s, 50 m/s, 100 m/s, and 150 m/s. Scribing distances of 30 Å, 60 Å, and 100 Å were also simulated. Initially, the diamond probe was positioned above the single-crystal silicon substrate at coordinates *x* = 300 Å, *y* = 0 Å, and *z* = 160 Å, with an initial tip-surface separation of 10 Å. Before scribing, the probe was lowered by 1 nm onto the substrate to establish contact, i.e., the indentation and scribing steps. Wear of the diamond probe under different simulation conditions was analyzed, and the specific conditions are summarized in Table 5.

## 3. Results and Discussion

### 3.1. Analysis of Test Results

#### 3.1.1. Analysis of Test Results at Different Scratch Speeds

**Figure 5:**
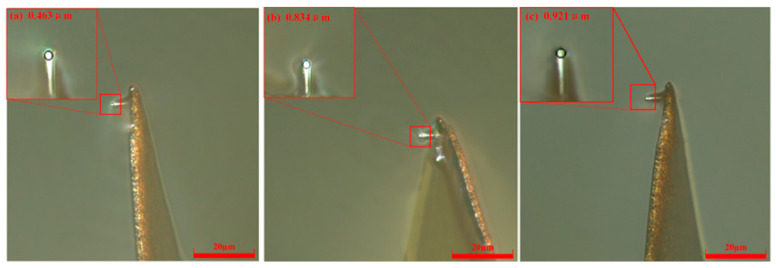
Probe radii with different scribing distances at *V* = 5 μm/s and *H* = 50 nA. (**a**) *L* = 60 μm; (**b**) *L* = 180 μm; (**c**) *L*= 300 μm.

A constant bias current of 50 nA and a uniform lateral scribing velocity of 5 μm/s were applied throughout the experiment. Figure 5 summarizes the evolution of the probe tip radius after cumulative scribing distances of 60, 180, and 300 μm. Specifically, the radius was measured as 0.463 μm after 60 μm, rose to 0.834 μm after 180 μm, and reached 0.921 μm following 300 μm of continuous scribing. This consistent radial expansion demonstrates progressive tip blunting, confirming that wear severity escalates with increasing scribing length.

**Figure 6:**
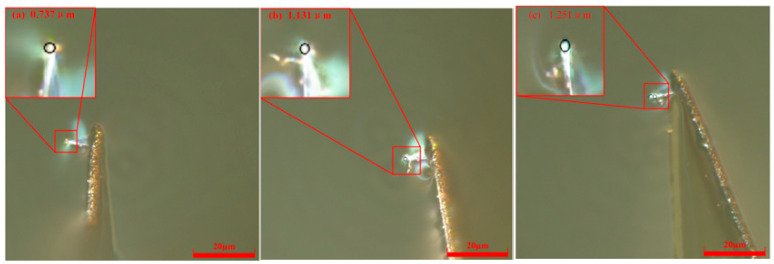
Probe radii with different scribing distances at *V* = 52.5 μm/s and *H* = 50 nA. (**a**) *L* = 60 μm; (**b**) *L* = 180 μm; (**c**) *L* = 300 μm.

**Figure 7:**
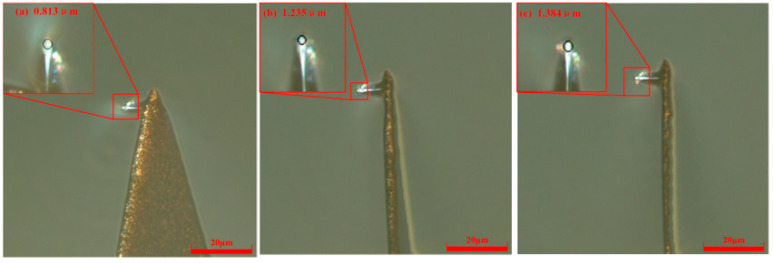
Probe radii with different scribing distances at *V* = 100 μm/s and *H* = 50 nA. (**a**) *L* = 60 μm; (**b**) *L* = 180 μm; (**c**) *L* = 300 μm.

Under a fixed bias current of 50 nA but with elevated scribing velocities (52.5 μm/s and 100 μm/s), the evolution of the diamond probe tip radius was systematically tracked at cumulative scribing distances of 60, 180, and 300 μm. As presented in Figure 6, at 52.5 μm/s, the measured radii were 0.737 μm (60 μm), 1.131 μm (180 μm), and 1.251 μm (300 μm). At the higher speed of 100 μm/s, which is shown in Figure 7, five repeated measurements were performed at each distance to ensure statistical robustness; the averaged tip radii were 0.813 μm, 1.235 μm, and 1.384 μm, respectively. Across both speed conditions, a monotonic increase in radius with scribing length is consistently observed, indicating progressive tip blunting and accelerated wear accumulation under faster scanning.

**Figure 8:**
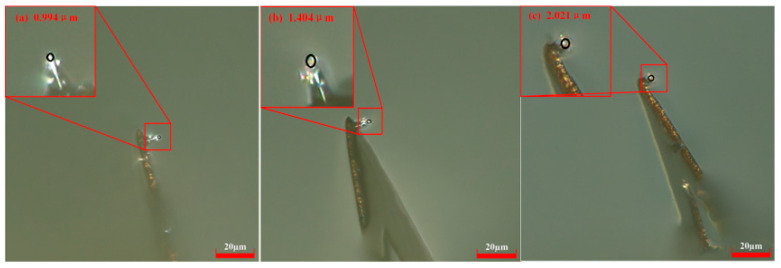
Probe radii with different scribing distances at *V* = 200 μm/s and *H* = 50 nA. (**a**) *L* = 60 μm; (**b**) *L* = 180 μm; (**c**) *L* = 300 μm.

At a scribing speed of 200 μm/s and constant current of 50 nA, the probe radius increased from 0.994 μm at 60 μm to 1.404 μm at 180 μm and 2.021 μm at 300 μm (Figure 8). The probe radius grew very quickly with increasing scribing distance. For instance, when the scribing distance changed from 60 μm to 300 μm, the radius increased from 0.994 μm to 2.021 μm. The experiments show that a scribing speed of 200 μm/s greatly accelerates probe wear.

**Figure 9:**
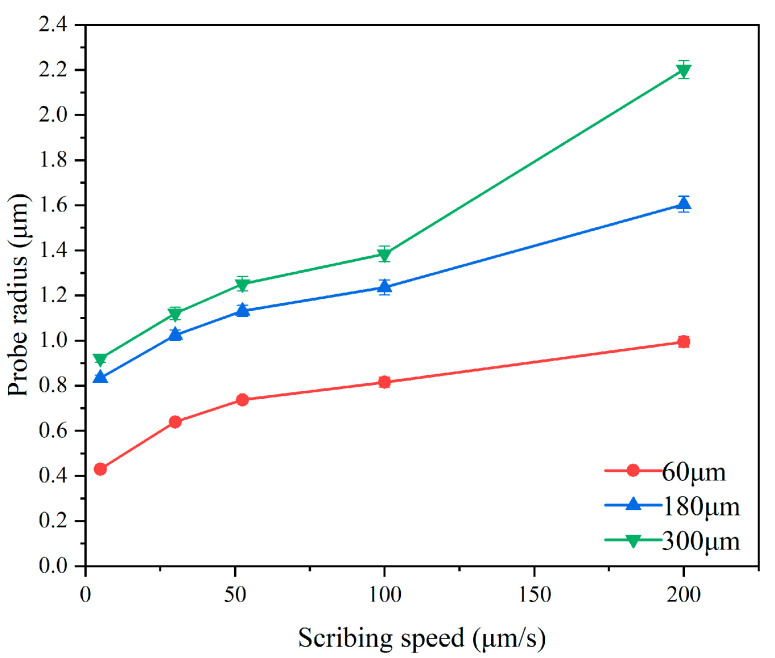
Variation of tip radius with scribing speed. Error bars represent standard deviation (n = 5).

In the single-factor tests, the control current was fixed at 50 nA, and scribing was performed at various speeds. The results show that at scribing distances of 60, 180, and 300 μm, the probe radius increases with scribing speed, indicating more severe wear at higher speeds. Under different scribing speeds, the change of probe wear at the same scribing distance is shown in Figure 9.

The experimental data in Figure 9 show that the probe radius increases significantly with increasing scribing speed and distance. When the scribing speed reaches 200 μm/s, the probe radius reaches 2.021 μm, and nonlinear growth occurs at this time.

#### 3.1.2. Analysis of Test Results Under Different Control Currents

**Figure 10:**
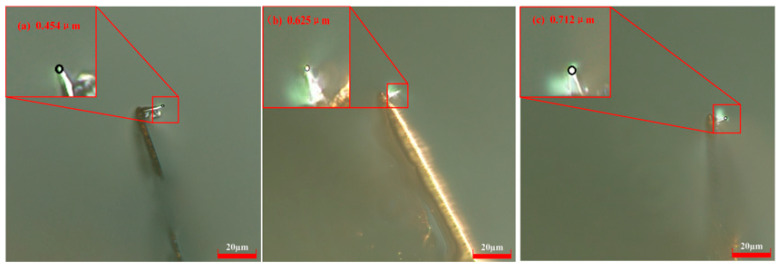
Probe radii at different scribing distances under *V* = 52.5 μm/s and *H* = 15 nA. (**a**) *L* =60 μm; (**b**) *L* = 180 μm; (**c**) *L* = 300 μm.

The experiment was carried out at a scribing velocity of 52.5 μm/s with a probe bias current of 15 nA. Tip radius measurements obtained via high-resolution digital microscopy revealed progressive blunting: 0.454 μm after 60 μm, 0.625 μm after 180 μm, and 0.712 μm following 300 μm of continuous scribing. Representative micrographs capturing this morphological evolution are presented in Figure 10.

**Figure 11:**
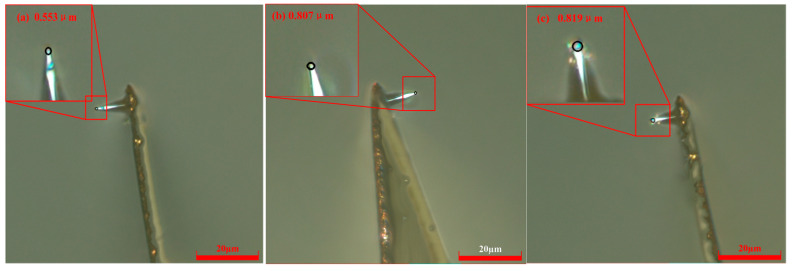
Probe radii with different scribing distances at *V* = 52.5 μm/s and *H* = 20 nA. (**a**) *L* = 60 μm; (**b**) *L* = 180 μm; (**c**) *L* = 300 μm.

When the scribing speed was 52.5 μm/s and the control current was 15 nA, the probe radius gradually increased with scribing distance. Using the same scribing speed of 52.5 μm/s and a control current of 20 nA, the measured probe radii were 0.553 μm at a scribing distance of 60 μm, 0.807 μm at 180 μm, and 0.819 μm at 300 μm. The digital microscope images are shown in Figure 11.

**Figure 12:**
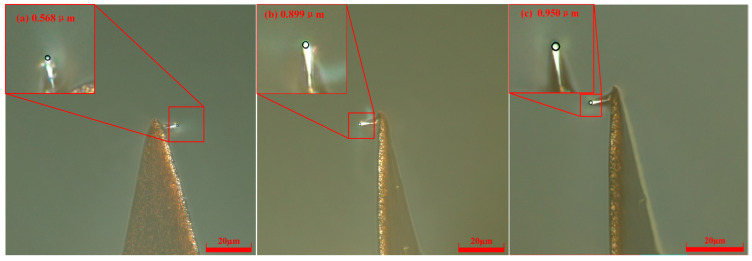
Probe radii at different scribing distances under *V* = 52.5 μm/s and *H *= 35 nA. (**a**) *L* = 60 μm; (**b**) *L* = 180 μm; (**c**) *L* = 300 μm.

At a constant scribing speed of 52.5 μm/s, single-factor experiments were conducted to study probe wear under control currents of 20 nA, 35 nA, and 50 nA. For a control current of 20 nA, the measured tip radii were 0.553 μm (60 μm), 0.807 μm (180 μm), and 0.819 μm (300 μm). At 35 nA, the radii increased to 0.568 μm, 0.899 μm, and 0.950 μm, respectively; at 50 nA, they further increased to 0.737 μm, 1.131 μm, and 1.251 μm. These results are shown in Figure 12, which presents the corresponding digital microscopy images.

**Figure 13:**
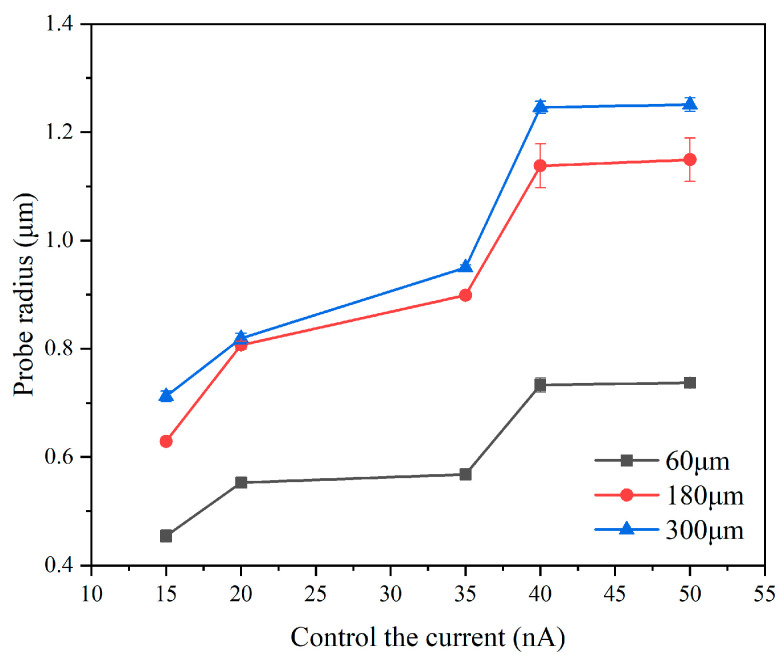
Variation of tip radius with control current. Error bars represent standard deviation (n = 5).

At a constant scribing speed of 52.5 μm/s, the effect of control current on probe wear was examined. At control currents of 50 nA, the measured probe wear radii were 0.737 μm, 1.131 μm, and 1.251 μm at scribing distances of 60 μm, 180 μm, and 300 μm. Similarly, at a control current of 40 nA, the reported wear radii were 0.733 μm, 1.120 μm, and 1.246 μm at these scribing distances. The relationship between tip radius and control current at 52.5 μm/s is summarized in Figure 13. It can be seen that at a given scribing speed, the probe wear radius increases with control current, indicating more severe wear at higher currents.

Scribing was performed at a constant velocity of 52.5 μm/s. Under a probe bias current of 50 nA, the tip radius increased from 0.737 μm after 60 μm of scribing to 1.131 μm at 180 μm and further to 1.251 μm at 300 μm, demonstrating cumulative wear with distance. Crucially, comparing these results with those obtained at the same speed but lower current, a clear trend emerges: elevated bias current intensifies tip degradation, resulting in significantly larger radii across all scribing lengths. This confirms that electrical loading plays a decisive role in accelerating diamond probe wear under identical mechanical conditions.

At a scribing speed of 52.5 μm/s and a control current of 40 nA, the measured tip radii were 0.733 μm (60 μm), 1.12 μm (180 μm), and 1.246 μm (300 μm). This result shows little difference compared with that obtained at a current of 50 nA. Additionally, it can be seen that the difference in tool wear condition between scribing distance of 180 μm and 300 μm is small.

#### 3.1.3. Analysis of Test Results at Different Scribing Distances

**Figure 14:**
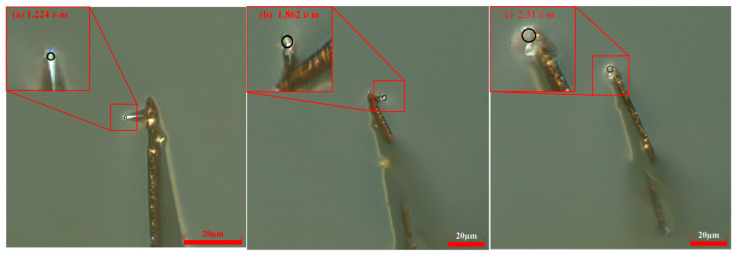
Probe radii at different scribing distances under *L* = 90 μm and *H* = 50 nA. (**a**) *V*= 5 μm/s; (**b**) *V* = 52.5 μm/s; (**c**) *V* = 100 μm/s.

Scribing distances of 60, 90, 180, 300, and 900 μm were tested. Results for a scribing distance of 90 μm (control current: 50 nA, scribing speed: 5 μm/s) under various cutting speeds are shown in Figure 14. Probe wear accelerated as scribing speed increased.

**Figure 15:**
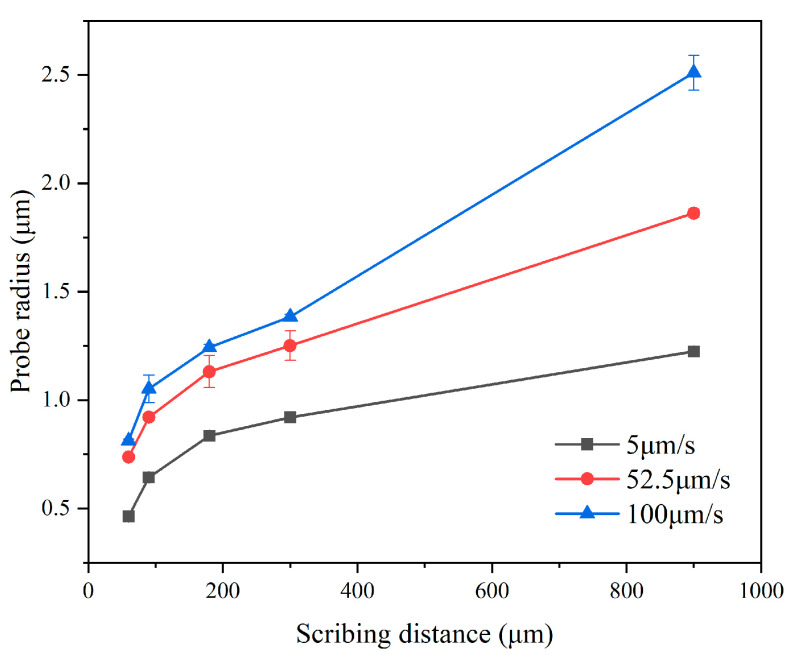
Variation of tip radius with scribing distance. Error bars represent standard deviation (n = 5).

For a control current of 50 nA, scribing speeds of 5, 52.5, and 100 μm/s were applied. The probe radius for each group was plotted against the scribing distance, as shown in Figure 15. Under a fixed control current, the probe radius increased nonlinearly with increasing scribing speed and distance. Moreover, the variation in wear radius is less pronounced in the 52.5 μm/s–100 μm/s speed range than in the 5 μm/s–52.5 μm/s range. At a scribing distance of 900 μm, the probe radius increased from 1.224 μm to 2.51 μm when the scribing speed increased from 5 μm/s to 100 μm/s.

When the velocity surpasses 52.5 μm/s, a pronounced increase in tip radius is observed—indicative of accelerated tip degradation, thereby exacerbating tip damage. To mitigate tip wear, experimental findings suggest adopting either a segmented scribing strategy or restricting the scribing speed to ≤52.5 μm/s for scan lengths within 300 μm.

#### 3.1.4. Verification of the Accuracy of Probe Tip Radius Measurement

**Figure 16:**
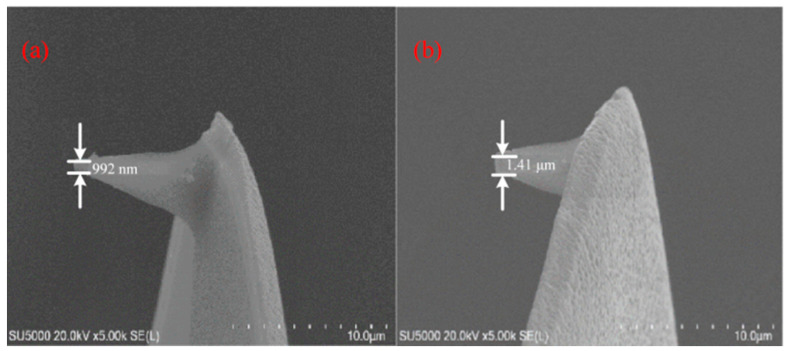
SEM micrographs of the probe at conditions of (**a**) *V* = 5 μm/s, *H* = 50 nA (**b**) *V* = 100 μm/s, *H* = 50 nA.

In order to verify the accuracy of the probe tip radius measurement by digital optical microscope, Figure 16 presents the representative micrograph results of probe tips after scribing over a fixed distance of 300 μm under *V* = 5 μm/s, *H* = 50 nA and *V* = 100 μm/s, *H* = 50 nA, respectively. It can be seen that the measurement results under conditions of *V* = 5 μm/s and 100 μm/s with the same current levels 50 nA are 0.992 μm and 1.41 μm. Compared with the results measured by digital optical microscope, the errors are within 7.2%, where it can be considered that the measurement data is basically accurate and reliable, taking into account the influence of human measurement factors.

### 3.2. Analysis of Simulation Results

#### 3.2.1. Simulation Analysis at Different Downward Pressure Depths

The simulation models the indentation process of a diamond tip on a single-crystal silicon surface, which proceeds through three stages: indentation, elastic deformation, and plastic deformation. During this initial stage, the probe is lowered to an indentation depth of 1 nm, establishing contact between the diamond tip and the silicon surface. Simulations were performed at indentation depths *H* of 10 Å, 15 Å, 20 Å, 25 Å, and 30 Å to observe wear evolution at each setting. Upon contact, stress is generated in the probe by the silicon surface. Various penetration depths induce different stress magnitudes and distribution, different degrees of elastic and plastic deformation of the probe. Based on the simulation results, probe profile evolution can be analyzed conveniently.

**Figure 17:**
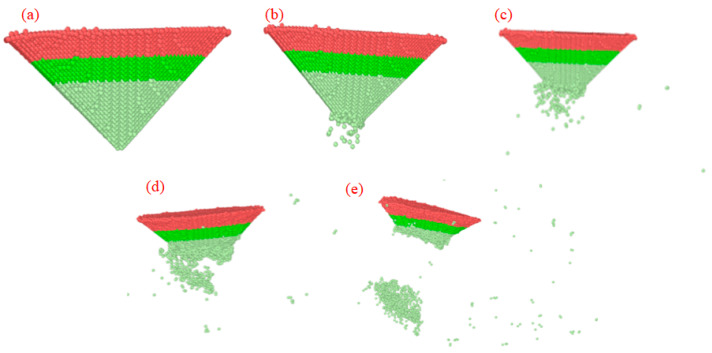
Simulation of diamond probe at different indentation depths. (**a**) *H* = 10 Å; (**b**) *H* = 15 Å; (**c**) *H* = 20 Å; (**d**) *H* = 25 Å; (**e**) *H* = 30 Å.

Figure 17 presents molecular dynamics simulation outcomes illustrating how diamond probe wear evolves with increasing normal indentation depth. At 10 Å, the probe undergoes purely reversible elastic deformation, no atomic loss or surface damage is observed. Upon raising the depth to 15 Å, localized bond rupture initiates at the tip apex, leading to detachment of several carbon atoms that subsequently adhere to the silicon substrate—an early but distinct signature of wear onset. At 20 Å, atomic ejection intensifies, and measurable material removal from the probe becomes evident. Further increasing the depth to 25 Å induces surface microcracking and widespread atomic shedding, signaling a transition toward irreversible damage. Complete structural failure characterized by macroscopic fracture and permanent plastic collapse occurs at 30 Å. Collectively, these results reveal a progressive shift in mechanical response: from elasticity to incipient wear to ductile-like atom removal to brittle cracking to catastrophic fracture as contact pressure rises with indentation depth. Notably, 15 Å marks the threshold where wear becomes detectable at the atomic scale without compromising probe integrity; thus, it represents a viable upper limit for stable, high-fidelity nano-scribing operations.

The simulations reveal adhesive wear primarily occurs through the exchange of atoms across the diamond–silicon interface. This is a phenomenon driven by the interfacial bonding behavior modeled by Tersoff potential.

When subjected to high normal loads, surface atoms at the contact region undergo significant local distortion and partial coordination loss. This enables short-lived C–Si bonds to form. These transient linkages cause atoms to detach from their original lattice sites and become incorporated into the opposing surface. This dynamic is commonly observed in experimental nanoscratching of silicon with diamond tips.

While this atomic shuffling mirrors real-world adhesive wear mechanisms in covalent systems, it results from the effective, environment-dependent bond-order formalism of Tersoff potential, rather than from explicit modeling of electron sharing or orbital hybridization.

**Figure 18:**
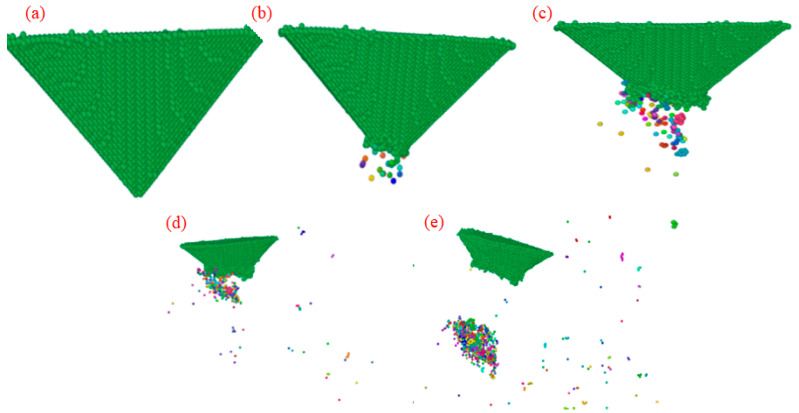
Cluster analysis of diamond tip wear at different indentation depths. (**a**) *H* = 10 Å; (**b**) *H*= 15 Å; (**c**) *H* = 20 Å; (**d**) *H* = 25 Å; (**e**) *H* = 30 Å.

Thus, the process is interpreted as a physically realistic proxy for adhesion-driven material transfer, constrained by the potential’s parametric scope. The cluster changes of the simulation results under different indentation depths are shown in Figure 18.

MD simulations reveal a systematic increase in cluster formation, comprising detached carbon atoms from the diamond probe, as indentation depth rises. At 10 Å, no clusters are observed, confirming purely elastic contact with no atomic ejection. At 15 Å, 28 clusters emerge, predominantly localized near the probe’s leading edge, indicating the onset of localized tip degradation. This number surges to 115 at 20 Å, with pronounced spatial asymmetry: clusters concentrate heavily at the front of the probe, reflecting directional material removal under forward scanning. At 25 Å, the cluster count climbs to 341, and their spatial distribution broadens significantly, spreading beyond the immediate contact zone and suggesting more extensive atomic displacement. By 30 Å, cluster generation peaks at 742, widely dispersed across the simulation domain; many are fully ejected into the surrounding space, and concurrent fracture of the probe tip is evident, accompanied by severe atomic rearrangement and plastic accumulation at the crack initiation site. Overall, cluster quantity serves as a quantitative proxy for wear severity; the monotonic rise in cluster count with indentation depth directly correlates with escalating structural damage from initial atom loss to catastrophic tip failure.

**Figure 19:**
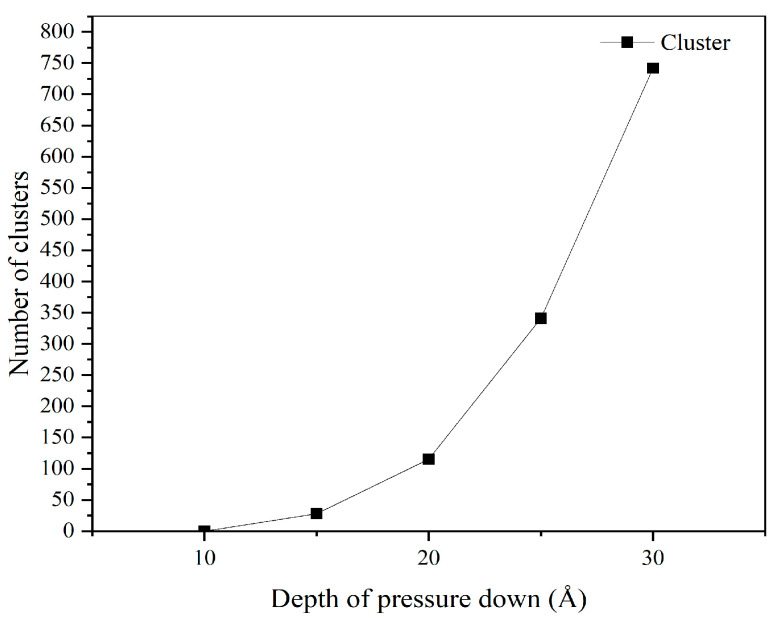
Change in tip wear based on cluster analysis with indentation depth.

Figure 19 shows the evolution of cluster count with indentation depth, revealing a monotonic increase across the tested range (10–30 Å). This trend indicates that increased indentation depth promotes atomic-scale disintegration of the diamond probe, providing qualitative insight into the relationship between mechanical loading and wear severity. Cluster count functions not only as a sensitive wear indicator but also as a scalable metric reflecting the severity of tip degradation under increasing normal load.

**Figure 20:**
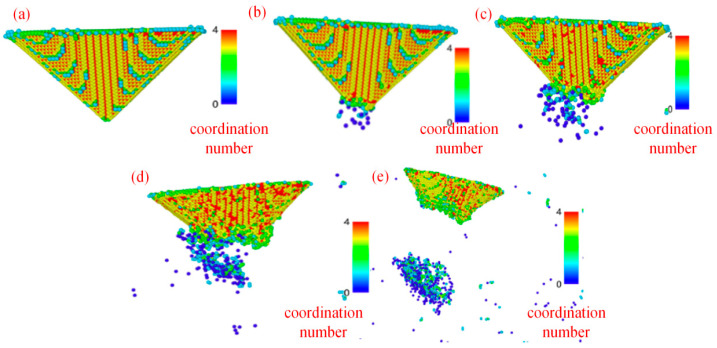
Coordination number analysis of diamond probes at different indentation depths. (**a**) *H* = 10 Å; (**b**) *H* = 15 Å; (**c**) *H* = 20 Å; (**d**) *H* = 25 Å; (**e**) *H* = 30 Å.

Figure 20 illustrates the evolution of atomic coordination numbers at the diamond probe tip during indentation-induced wear. In an ideal diamond lattice, each carbon atom maintains four covalent bonds (coordination number = 4) in a tetrahedral configuration. As mechanical loading intensifies, bond rupture occurs—particularly at the apex—leading to under-coordinated surface atoms with coordination numbers of 0–3. These atoms are visually distinguished in the simulation by distinct color coding. Notably, a reduction from CN = 4 to CN = 3 is observed, hinting at possible bond hybridization changes, though further analysis and definitive phase identification requires complementary analysis. Quantitative tracking (Table 6) shows that with increasing indentation depth, the population of CN = 4 atoms steadily declines, while the count of CN = 3 atoms rises monotonically. This progressive loss of tetrahedral coordination directly correlates with escalating tip degradation, positioning CN = 3 as a robust, atomistically resolved marker of incipient graphitization and wear progression.

**Figure 21:**
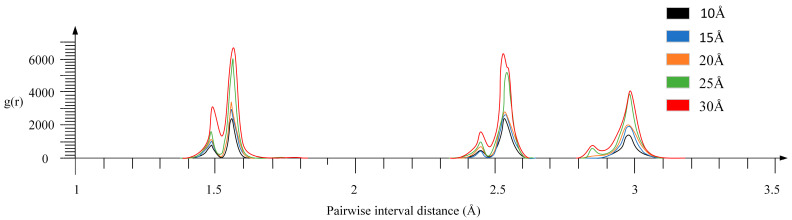
RDF analysis of diamond probes at different depths of indentation.

The radial distribution function (RDF), also referred to as the pair distribution function (PDF), is a fundamental statistical descriptor in molecular dynamics simulations, quantifying the probability of finding a particle at a given distance from a reference particle. In this study, RDF analysis was applied to the diamond probe to assess structural integrity and detect potential graphitization—i.e., the local transformation of sp^3^-bonded diamond into sp^2^-bonded graphite-like phases—under mechanical compression. Simulations were conducted at multiple indentation depths, and RDFs were computed from the equilibrated atomic configurations. The resulting RDF profiles are presented in Figure 21.

Based on RDF, the degree of diamond graphitization during the friction process was further analyzed, as shown in Figure 21. The bonds between diamond atoms consist of sp^3^ bonds, and the C–C bond length between diamond atoms is 1.54 Å, while the secondary nearest-neighbor distance between diamond atoms is 2.52 Å. The bonds between graphite atoms consist of sp^2^ bonds, with the C–C bond length being that of the sp^2^ bond, and the intralayer secondary nearest-neighbor distance is 2.46 Å. The interlayer peak is located at 3.35 Å.

When the compression depth is 10 Å, there is no obvious peak at 1.42 Å, and the main peak is located at 1.54 Å. Here, the peak is the peak of diamond atoms, and the secondary peak is obvious at 2.52 Å, indicating that the secondary nearest-neighbor structure of diamond is intact. At this time, the diamond probe has not undergone graphitization, and the structure remains a sp^3^ hybridization. When the compression depth is 15 Å, a weak peak appears at 1.42 Å, and the 1.54 Å peak slightly rises. This is due to the disorder of bond lengths caused by local stress. At this time, sp^2^ bonds begin to appear, but no long-range ordered bonds are formed. When the compression depth is 20 Å, the peak at 1.42 Å significantly increases and coexists with the 1.54 Å peak. A new peak appears at 2.46 Å, which corresponds to the secondary nearest neighbor within the graphite layer. At this time, the proportion of sp^2^ bonds increases, indicating a transition to the graphite phase. When the depth reaches 25 Å and 30 Å, the peak at 1.42 Å further increases, and the 2.46 Å peak persists. This indicates that sp^2^ bonds and graphite-like structures continue to develop. The diamond characteristic peak at 1.54 Å still dominates, and there is no peak at the 3.35 Å graphite interlayer distance at all depths. This indicates that even at the maximum compression depth of 30 Å, the main structure of the diamond probe is still dominated by sp^3^-hybridized diamond, indicating a local graphitization transformation, but not complete graphitization.

When the compression depth is no more than 15 Å, there is basically no graphitization; after 20 Å, local graphitization occurs, and the degree of graphitization increases with the increase in depth; but at the 30 Å depth, the structure is still dominated by the diamond phase, with local graphitization.

#### 3.2.2. Simulation Analysis at Different Scribing Speeds

Analysis of the simulation results at different indentation depths shows that when the indentation depth is 10 Å, the diamond probe undergoes elastic deformation without wear. At this point, when the probe becomes a scribing tool, its wear rate is the lowest, thus making it an ideal parameter for scribing. Nevertheless, from the wear track of the parameter studied, this parameter is also not suitable for studying diamond probe wear. At indentation depth 15 Å and 20 Å, wear begins at the bottom of the diamond probe without fracture. As indentation continues to increase, the fracture becomes significant at the bottom of the diamond probe. Because of the overall removal effect of the diamond probe on single-crystal silicon material, the indentation depth is chosen to be 15 Å as a fixed variable for later simulations, and single-factor simulations are conducted on the scribing speed and distance.

**Figure 22:**
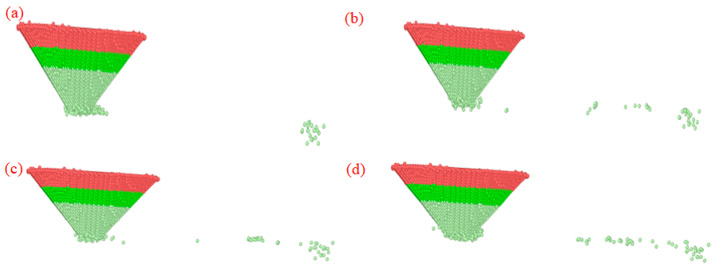
Simulation analysis of diamond probe under different scribing speeds. (**a**) *V* = 50 m/s; (**b**) *V* = 100 m/s; (**c**) *V* = 150 m/s; (**d**) *V* = 200 m/s.

MD simulations reveal a clear velocity-dependent wear behavior of the diamond probe during nanoscale scribing of monocrystalline silicon, as shown in Figure 22. At 50 m/s, atomic-scale inspection after 100 Å of travel shows three distinct wear phases: initial detachment of surface carbon atoms from the probe tip upon first contact; a transient stabilization phase with negligible atom loss; and late-stage redeposition, where dislodged diamond atoms are swept backward by shear forces and accumulate near the probe’s trailing edge. At 100 m/s, wear initiates rapidly at the apex during early scribing but progressively attenuates as scanning continues, suggesting dynamic passivation or redistribution of stress. Raising the speed to 150 m/s amplifies both the magnitude and spatial extent of tip blunting, with greater radial expansion compared to 100 m/s. At 200 m/s, the most severe degradation occurs: the probe radius increases markedly, and worn carbon atoms undergo substantial plastic rearrangement, forming a deformed, atomically mixed layer around the enlarged tip region. Mechanistically, wear is driven by shear-induced bond scission between diamond atoms, followed by re-adhesion, either to the silicon substrate or to neighboring carbon sites on the probe surface characteristic of adhesive wear. This process intensifies with velocity due to elevated thermal and mechanical energy input, culminating in the most pronounced adhesion-mediated degradation at 200 m/s. Collectively, these findings establish scribing speed as a dominant kinetic parameter governing both wear rate and morphology evolution.

**Figure 23:**
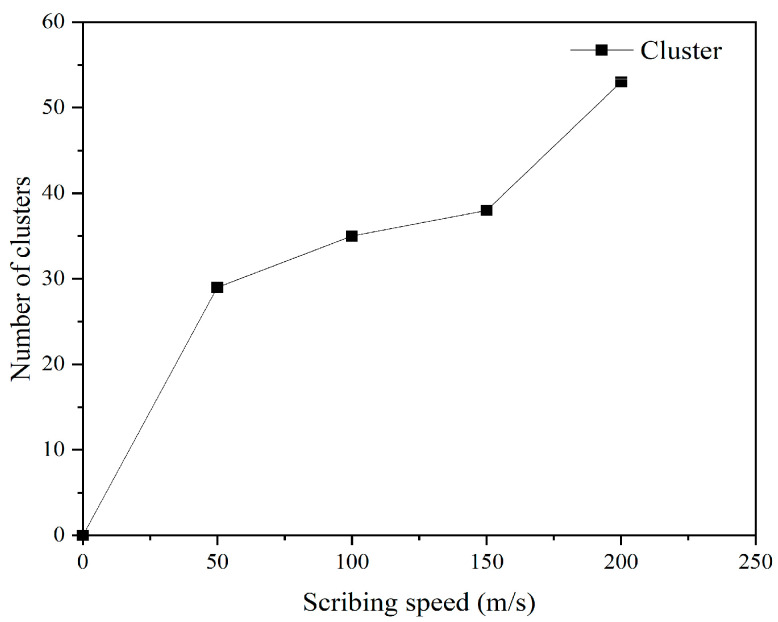
Variation of the number of clusters in the diamond tip with scribing velocity.

The conditions of indentation depth and the scribing distance were fixed at 15 Å and 100 Å, and cluster analysis of the results showed that the number of atomic clusters produced increased with scribing speed: 29 clusters at 50 m/s, 35 clusters at 100 m/s, 38 clusters at 150 m/s, and 53 clusters at 200 m/s. This trend suggests that higher scribing velocity promotes atomic detachment, consistent with increased energy input at the interface. The relationship between scribing speed and cluster count is shown in Figure 23.

#### 3.2.3. Simulation Analysis at Different Scribing Distances

**Figure 24:**
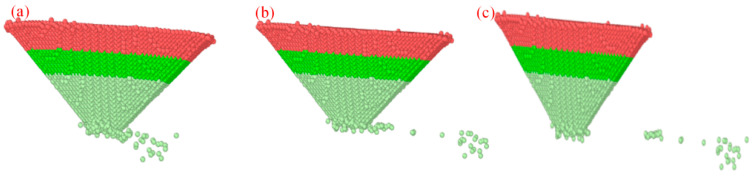
Diamond tip simulation at different scribing distances. (**a**) *L* = 30 Å; (**b**) *L* = 60 Å; (**c**) *L =* 100 Å.

During scribing, the probe exhibits different wear phenomena at different scribing distances. The simulation used an indentation depth of 15 Å and a scribing speed of 100 m/s to analyze the wear process at scribing distances of 30 Å, 60 Å, and 100 Å, as shown in Figure 24.

At a scribing distance of 30 Å, the applied downward force begins to detach diamond atoms from the probe tip. During the initial scribing stage, detached diamond atoms are transferred and accumulate on the single-crystal silicon surface due to scribing forces. As the scribing distance increases, the number of diamond atoms ejected decreases. Friction at the scribing interface also begins to break bonds between carbon atoms in the probe tip. A few diamond atoms detach onto the single-crystal Si surface, while the remaining, more strongly bonded atoms at the tip undergo slight deformation.

**Figure 25:**
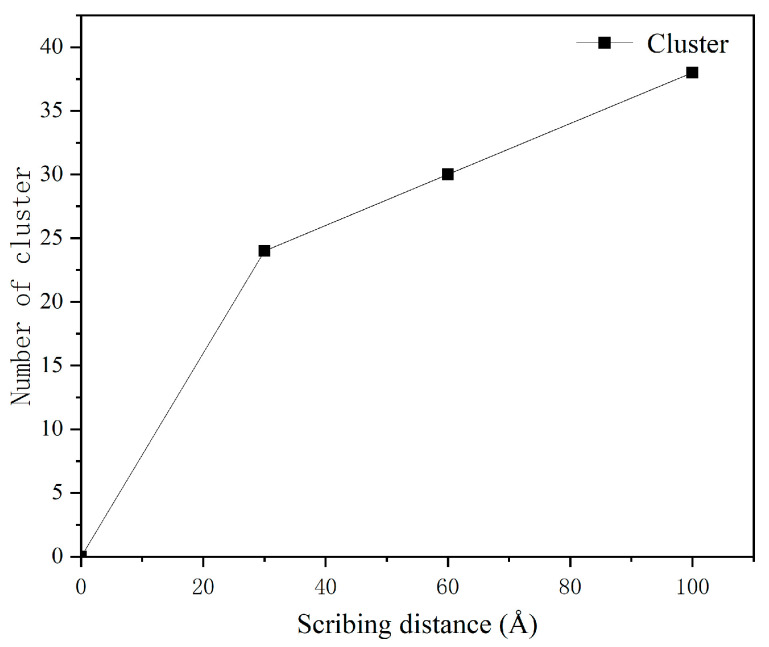
Changes in cluster count at different scribing distances.

Cluster analysis was performed at an indentation depth of 15 Å and a scribing speed of 100 m/s for scribing distances of 30 Å, 60 Å, and 100 Å. At a scribing distance of 30 Å, 24 clusters were produced; at 60 Å, 30 clusters; and at 100 Å, 38 clusters. This increase indicates that prolonged sliding promotes cumulative atomic detachment, consistent with the progression of adhesive wear observed in the simulations. The variation of the cluster number with scribing distance is shown in Figure 25.

## 4. Conclusions

This study investigates the wear mechanism of diamond probes during scribing of single-crystal silicon by combining scribing experiments with molecular dynamics simulations. While MD simulations employ idealized conditions with high strain rates, they provide valuable atomic-scale insights that complement the experimental observations. The main findings are as follows:

(1) Experimental findings demonstrate that quantitative analysis reveals a clear positive correlation between tip wear severity and three key process parameters: applied electrical current, scanning velocity, and total scratching distance. Molecular dynamics simulations also indicate that atomic attrition contributes to material loss.
(2) A fully atomistic MD simulation framework was developed in LAMMPS to model the nanoscale interaction between a diamond probe and a monocrystalline silicon substrate. The simulations varied indentation depth, scribing velocity, and scribing distance. The results show that probe wear, quantified by tip radius, atomic detachment, cluster formation, and coordination number reduction, increases monotonically with each parameter. Adhesive wear, characterized by carbon–silicon interfacial bonding and carbon–carbon reattachment, occurred in all simulated conditions. This confirms its key role in diamond probe degradation during nanoscribing.
(3) As indentation depth increases, more atoms detach from the probe tip, leading to atom ejection. The number of atoms that transition from coordination number 4 to 3 also increases with the depth increasing, contributing to wear. At depths above 25 Å, wear changes from elastic to plastic deformation; at 30 Å, the tip deforms plastically and fractures. As scribing speed increases, atomic-level wear dominates in the early stage. With further increases in speed, the number of wear-generated clusters grows, and probe wear accelerates faster than the scribing speed. At an indentation depth of 15 Å and a scribing speed of 100 m/s, the number of detached diamond atoms increases with scribing distance.

## Figures and Tables

**Table 1:** Relationship between control current and actual load.

Control Current (nA)	Measured Load (μN)
10	4.68 ± 0.19
20	9.37 ± 0.37
30	14.05 ± 0.56
40	18.73 ± 0.75
50	23.42 ± 0.94

**Table 2:** Experimental design.

Scribing Velocity (μm/s)	Control Current (nA)	Scribing Distance (μm)
5	50	60, 180, 300
30	50	60, 180, 300
52.5	50	60, 180, 300
100	50	60, 180, 300
200	50	60, 180, 300
52.5	15	60, 180, 300
52.5	20	60, 180, 300
52.5	35	60, 180, 300
52.5	40	60, 180, 300
52.5	50	60, 180, 300
5, 52.5, 100	50	60
5, 52.5, 100	50	90
5, 52.5, 100	50	180
5, 52.5, 100	50	300
5, 52.5, 100	50	900

**Table 3:** Parameters of the diamond probe model.

Parameter	Value
Probe material	Diamond
Probe shape	Solid cone
Number of probe atoms	23,302
Probe tip angle (°)	90
Height (Å)	50

**Table 4:** Model parameters of monocrystalline silicon sample.

Parameter	Value
Sample material	Monocrystalline silicon
Sample shape	Cuboid
Number of sample atoms	123,981
Sample surface orientation	[100] (*100*)
Sample dimensions (Å)	300 × 90 × 150

**Table 5:** Simulation parameters.

Name	Parameter
Probe material	Diamond
Workpiece material	Monocrystalline silicon
Probe shape	Cone
Substrate dimensions (*X* × *Y* × *Z*)(Å)	300 × 90 × 150
Indentation depth (Å)	10, 15, 20, 25, 30
Scribing velocity (m/s)	10, 50, 100, 150
Scribing distance (Å)	30, 60, 100
Initial temperature (K)	298
Total number of atoms	147,283

**Table 6:** Changes in diamond coordination number at different depths of indentation.

Indentation Depth/Å	Count of 3-Fold Coordinated Atoms	Number of Atoms Transformed to Coordination Number 4
10	0	0
15	32	106
20	187	481
25	1648	2349
30	6360	8047

## Data Availability

The original contributions presented in this study are included in the article. Further inquiries can be directed to the corresponding authors.
